# The subcutaneous movements of filarial infective larvae are impaired in vaccinated hosts in comparison to primary infected hosts

**DOI:** 10.1186/1475-2883-4-3

**Published:** 2005-05-25

**Authors:** Simon A Babayan, Tarik Attout, Phat N Vuong, Laetitia Le Goff, Jean-Charles Gantier, Odile Bain

**Affiliations:** 1 Parasitologie Comparée et Modèles Expérimentaux, Muséum National d'Histoire Naturelle, Paris, France; 2 Institutes of Evolution, Immunology and Infection Research, University of Edinburgh, Edinburgh, UK; 3 Unité d'Anatomie et de Cytologie pathologiques, Hôpital St Michel, Paris, France; 4 Laboratoire de Parasitologie, Faculté de Pharmacie, Chatenay-Malabry, France

## Abstract

Our aim in this study was to observe the movements of filarial infective larvae following inoculation into the mammalian host and to assess the effect of vaccination on larval migration, *in situ*. Here we present recordings of larvae progressing through the subcutaneous tissues and inguinal lymph node of primary infected or vaccinated mice. We used the filaria *Litomosoides sigmodontis *in BALB/c mice that were necropsied 6 hours after the challenge inoculation of 200 larvae. Subcutaneous tissue sections were taken from the inoculation site and larvae were filmed in order to quantify their movements. Our analyses showed that the subcutaneous larvae were less motile in the vaccinated mice than in primary-infected mice and had more leucocytes attached to the cuticle. We propose that this reduced motility may result in the failure of a majority of larvae to evade the inflammatory reaction, thereby being a possible mechanism involved in the early vaccine-induced protection.

## Findings

Effective protection induced by vaccination with irradiated larvae has been assessed in all filarial systems studied [1-3]. Previous studies have also shown that a characteristic feature of the immune response generated by irradiated larvae vaccines is a strong Th2 polarization [4-8]. With the model *Litomosoides sigmodontis *in mice [9], protection has been shown to occur within the first 48 hours post-inoculation and that the killing of larvae takes place in the subcutaneous tissue as in primary infected mice [10]. However, whereas only neutrophils infiltrate this tissue in primary infected mice, eosinophils are also present in vaccinated mice, and in conjunction with B cells account for the protection [11]. In both primary-infected and vaccinated mice, the larvae that survive are those that penetrate into the lymphatic vessels [11-14].

Inflammatory processes, coupled or not with the Th2 bias, affect the cellular and extracellular composition of the subcutaneous tissue, thus modifying its physical properties [15-21]. Therefore, it appeared relevant to investigate the effects of vaccination on this early but decisive phase of the larvae's migration, more precisely, on the motility of the larvae passing through the connective tissues of the mice. Indeed, filarial worms, like their free-living ancestors, have a powerful musculature and sensorial organs consisting of amphids and head papillae that allow autonomous and directional movements. In two non-filarial nematode parasites, the motility has been studied in naive and immunized hosts, and a reduction assessed [22,23].

The analysis of the mechanical activity of filarial nematodes in subcutaneous tissues at the onset of infection required a tailored protocol.

*L. sigmodontis *infective larvae were obtained as previously described [24]. Six week-old BALB/c mice, from Charles River, Cléon, France, were vaccinated with three inoculations of irradiated infective larvae as previously described [11]. Mice were challenged with 200 infective larvae (L3) inoculated subcutaneously in the right lumbar region. All experiments and procedures conformed to the French Ministry of Agriculture regulations for animal experimentation (1987).

Six hours after the challenge inoculation, the mice were culled, their skin separated from the animal and placed inner side up on a dissection board. The inguinal and iliac lymph nodes were then sampled, placed between a microscope slide and cover slip, lightly crushed then observed under the microscope.

**Figure 1:**
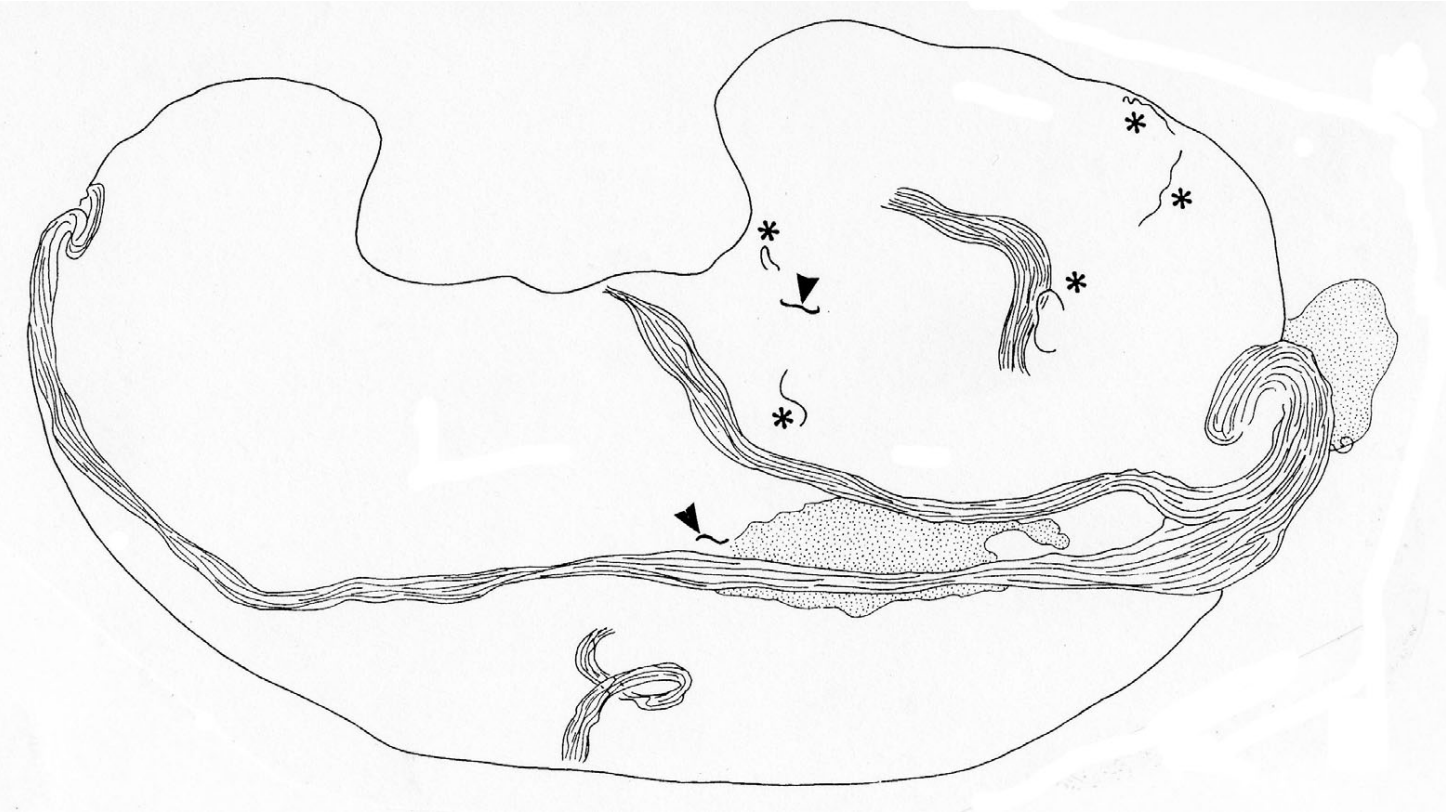
A depiction of the subcutaneous tissue of mice inoculated with *L. sigmodontis *observed 6 hours post challenge: thin tissue section mounted in RPMI 1640, with dispersed infective larvae (asterisks, or arrowhead if damaged by the razorblade), a few nerves and adipose cells (dotted area) are present.

A total of ten ~50 μm-thick slices of subcutaneous tissue were taken from the zone where the worms were inoculated with a razor blade, progressively spiraling away from that zone. The sections were then mounted for examination under a microscope, in a drop of RPMI 1640 (Fig. 1). Larvae that were found on the edge of the subcutaneous sample or cut by the razor blade were not included in the study. Each mouse was studied for no longer than an hour. Preparations that contained live worms were digitally recorded directly under the microscope, with Adobe Premiere ^® ^5.0 at 25 frames per second. Three magnifications were used: ×10, ×25 and ×100. Recordings lasted 30 seconds for each magnification and the movement of the worms assessed over that period with the following criteria: type of movements (undulating or uncoordinated), forward or backward progression, speed, oscillations of the head. The non-parametric Mann & Whitney's U test was used to compare groups.

Five primary infected mice and 5 vaccinated mice were necropsied 6 hours post-challenge. At the dissection microscope, the inoculated area was easily identified because the subcutaneous tissue had a gelatinous aspect, was swollen by oedema and was densely infiltrated by leukocytes particularly in vaccinated mice. A total of 2000 L3 were inoculated, of which 1.95 % were identified and filmed: 39 in the subcutaneous tissue and 4 in the inguinal lymph node. This low ratio of recovery indicates that the larvae were outside the area studied. Larvae extracted from lymph nodes were all from a primary-infected mouse. They undulated and sometimes progressed rapidly, though with apparently poor coordination (Additional file 1); however, good coordination was obtained when pressure was exerted on the cover slip.

The observations of infective larvae in subcutaneous tissues were reported according to the following criteria, which readily discriminate those in primary-infected from those in vaccinated mice: the intensity of movements, the undulations and progression of the worm's head, and host cells attached to or up against the larva. Table 1 gives details about the larvae filmed and the numbers of larvae in each category.

**Figure 2:**
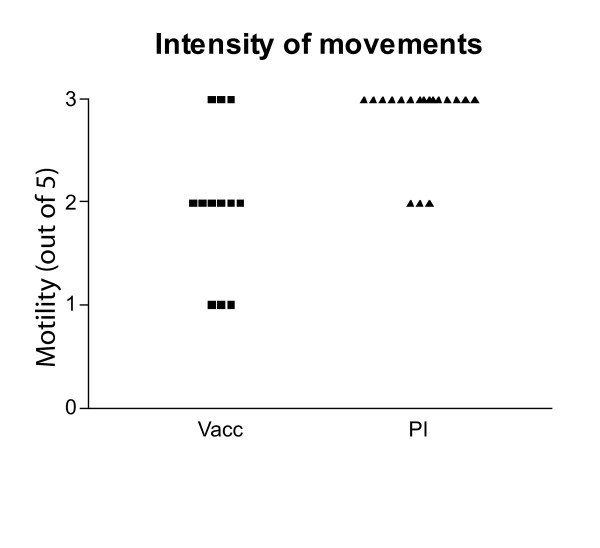
Assessements of the motility of infective larvae. The movements were assessed on recordings made of larvae in the subcutaneous tissue of vaccinated (Vacc) or primary infected (PI) mice and were scored on a discrete scale from 0 (non-motile) to 5 (maximum motility seen in larvae released in media only).

In both groups of mice, the subcutaneous tissue preparation in which larvae undulated had a gel-like consistency. In primary-infected mice the larvae's head oscillated from one side to another at an estimated angle of 20° from the body axis. The rest of the body undulated: well-coordinated S-shaped curves moved from head to tail when the larvae moved forward, and from tail to head when the larvae moved backwards (Additional file 2). The larvae were generally vigorous, often progressing forward in the subcutaneous tissues as observed in 74% of the worms studied. In this environment, the larvae pushed themselves forward by taking advantage of denser parts of the tissues (Additional file 2). In primary-infected mice their movements reached 2.87 on a scale of 5, corresponding to fast movements (Fig. 2). We managed to measure the distance some larvae covered: 8 mm in 30 seconds, corresponding to 1.5 cm per minute.

In vaccinated mice, the larvae's movements were reduced as compared to what had been observed in the primary infections (average of 2 on 5, Additional file 3). A convenient measure of the larvae's movements was the amplitude of their head's side-to-side movements (Additional file 4). Only 8% (1 out of 13 filariae) of the infective larvae moved their head forward in vaccinated mice whereas 73% (11 out of 15 filariae) did in primary-infected mice. Additionally, we more often observed cells attached to the larvae's cuticle, on the anterior third of the body (3 out of 17 worms vs. 1 out of 10, Additional files 3 and 5). Interestingly, we observed that these granulomas form first in the front third of the worm (Additional file 5).

In conclusion, this preliminary study shows that by vaccinating the hosts, the movements of the worms are significantly impaired in comparison to primary-infected hosts and that more cells attach to their cuticle, thus contributing to their killing. Therefore, we suggest that this would prevent or delay entry into the lymphatic vessels through which they evade the inflammatory reaction and migrate to the pleural cavity.

**Table 1:** Distribution of infective larvae (L3) observed in the subcutaneous tissues of vaccinated (Vacc, mice 1 to 5) and primary-infected (PI, mice 6 to 10) mice 6 hours after the challenge inoculation. The larvae that broke out of the tissue sample during the procedure were not included in the rest of the study.

**Mouse**	**Number of L3 in challenge**	**Number of larvae recovered**	**Number of non-motile or abnormal larvae**	**Number of larvae recorded**	**Number of larvae in sub cut tissue**	**Number of larvae escaped during procedure**	**Number of larvae in lymph node**
1	200	1	0	1	1	1	0
2	200	6	1	4	4	1	0
3	200	3	0	3	3	1	0
4	200	6	1	5	5	0	0
5	200	12	1	11	11	5	0

TOTAL VACC.	1000	28	3	24	24	8	0

6	200	4	4	0	0	0	0
7	200	5	2	3	3	2	0
8	200	8	1	7	3	1	4
9	200	7	1	0	0	0	0
10	200	13	4	9	9	1	0

TOTAL PI	1000	37	12	19	15	4	4

## Competing interests

The author(s) declare that they have no competing interests.

## Authors' contributions

SAB was responsible for carrying out the laboratory experiments described in the manuscript, including mouse preparation, dissections, sample processing and recording and writing the manuscript. TA and PNV carried out the histological studies. LL recorded the video sequences presented as additional files and JCG provided the microscope, software and hardware and his expertise for filming the preparations. OB is the Principal Investigator on the project, and was responsible for obtaining grant support for the project, for the experimental design, the data analysis and the preparation of the manuscript.

## Acknowledgements

This study was supported by the European Community grant ICA4-CT-1999-10002.

## Supplementary Material

Additional File 1 Infective larva extracted from a lymph node crushed in RPMI media. The larva's movements are rapid and uncoordinated, as it has no grip on its immediate environment. Many cells (mostly lymphocytes) are present in the media around the worm. Magnification: × 100. Click here for file

Additional File 2 The infective larva in subcutaneous tissue has a well-coordinated, large-amplitude S-shaped movement by which it leans on the surrounding tissue to progress forward. The fibrous structure of the subcutaneous tissue is visible, with many infiltrated cells (mainly neutrophils). Magnification: × 200 Click here for file

Additional File 3 Larva from a vaccinated mouse: it has reduced movements with low amplitude oscillations and a slow forward progression. The tissue has a dense appearance, with many infiltrated cells some of which can be seen right against the cuticle of the worm. The end of the sequence shows the absence of head side-to-side oscillations. Magnification: × 100 and × 500. Click here for file

Additional File 4 Detailed recording of the head of an infective larva from a vaccinated mouse. The head is only slightly oscillating from side-to-side, and is trapped in a dense gel-like structure with few discrete elements. This larva will certainly not have reached a lymphatic vessel. Magnification: × 500. Click here for file

Additional File 5 Example of how cells and subcutaneous fibrous structures attach to the cuticle of infective larvae. In this preparation, the larva was nearly freed into the dissection media, but remained attached by granulomatous formations at two points, near the head and around the last third of the body. Its movements are rapid, nearly frantic. Magnification: × 200. Click here for file
